# Characterizing environmental and phenotypic associations using information theory and electronic health records

**DOI:** 10.1186/1471-2105-10-S9-S13

**Published:** 2009-09-17

**Authors:** Xiaoyan Wang, George Hripcsak, Carol Friedman

**Affiliations:** 1 Dept of Biomedical Informatics, Columbia University, New York 10032, USA

## Abstract

**Background:**

The availability of up-to-date, executable, evidence-based medical knowledge is essential for many clinical applications, such as pharmacovigilance, but executable knowledge is costly to obtain and update. Automated acquisition of environmental and phenotypic associations in biomedical and clinical documents using text mining has showed some success. The usefulness of the association knowledge is limited, however, due to the fact that the specific relationships between clinical entities remain unknown. In particular, some associations are indirect relations due to interdependencies among the data.

**Results:**

In this work, we develop methods using mutual information (MI) and its property, the data processing inequality (DPI), to help characterize associations that were generated based on use of natural language processing to encode clinical information in narrative patient records followed by statistical methods. Evaluation based on a random sample consisting of two drugs and two diseases indicates an overall precision of 81%.

**Conclusion:**

This preliminary study demonstrates that the proposed method is effective for helping to characterize phenotypic and environmental associations obtained from clinical reports.

## Background

The availability of up-to-date, executable, evidence-based medical knowledge is essential for clinical applications such as quality of care, decision support, clinical information needs and hypothesis generation. Traditional work in the field has focused on constructing and maintaining knowledge bases manually using expert medical resources, but it is costly and time-consuming to establish evidence-based knowledge [1,2]. Additionally, because medical knowledge constantly changes, updating the knowledge is also problematic. It is, therefore, beneficial to develop automated methods to help create evidence-based knowledge and to help keep it up to date.

**Figure 1:**
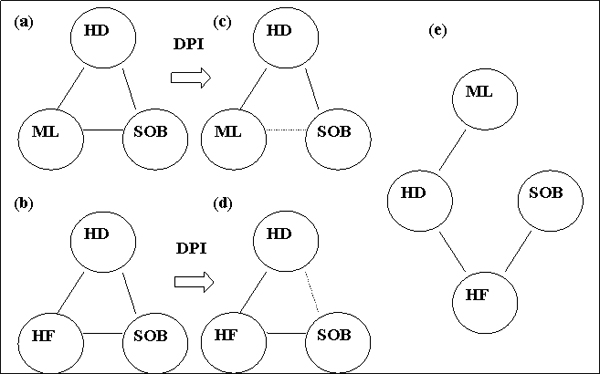
**Schematic diagram of the effect of DPI**. **(a) **and **(c)**: Among two paths between ML and SOB (ML-SOB, ML-HD-SOB), ML-SOB was eliminated by applying DPI (shown as 1 in table 1). **(b) **and **(d)**: Similarly, among two paths between HF and SOB (HF-SOB, HF-HD-SOB), HF-HD-SOB was eliminated by applying DPI (shown as 6 in table 1). **(e) **Combining evidence from (c) and (d), the path between ML-SOB would more likely to be ML-HD-HF-SOB. ML: Metolazone; HD: Hypertensive Disease; HF: Hear Failure; SOB: Shortness of Breath.

It is well known that much of evidence-based knowledge in biomedicine is buried in medical literature, reference websites and books, and narrative clinical reports [3-5]. In this work, we focus on narrative clinical reports. Generating associations, such as drug side effects, disease-drug therapies, and disease-symptom associations, based on phenotypic and environmental information in patient records provides knowledge concerned with the practice of medicine and the disease process, and acquisition of this knowledge has become possible with advances in natural language processing and data mining. Several lines of investigations have studied automated methods for creating and updating knowledge bases from narrative reports of patient records [6-9]. Those studies demonstrated that automated methods involving natural language processing (NLP) and statistics can generate meaningful associations between clinical phenotypes and drugs with great efficiency. Although these associations are useful, they have limitations because the specific relationships are not known but are critical. In particular, some associations are indirectly related. For example as shown in Figure 1a, using association statistics based on information in the patient record, we found that a symptom *shortness of breath *(SOB) was associated with a disorder *hypertensive disease *(HD). SOB was also found to be associated with a drug *Metolazone *(ML), and ML was found to be associated with HD. However, the statistical method was not able to determine whether the symptom SOB was directly associated with the drug ML and therefore caused by it or treated by it, or the symptom SOB was just indirectly associated with ML because it was the manifestation of the disease HD that it treats, and therefore was a confounding event. Characterizing the associations as 'direct' or 'indirect' would be an important feature for a medical knowledge base to have but resolving these confounding interdependencies is complicated.

Statistical adjustments have been widely used in many epidemiological studies in order to infer confounding effects of variables [10,11]. For example, people who drink more coffee may also smoke more cigarettes and drink more alcohol. Determining whether coffee drinking by itself increases mortality risk, and is not just a marker for some other causal factor can be approached using statistical methods [12]. The basic methods are to include measures of potential confounders as covariates in a regression model, or to stratify the data on these confounders. These methods are often called "statistically controlled" or "adjusted" for potential confounders.

While statistically adjusted methods provide a rich means of inferring confounding effects of variables, they still cannot characterize the associations or differentiate between those that are direct or indirect. Further research would be desirable to develop sophisticated models for this purpose.

Mutual information (MI), a measure of the degree of statistical dependence between two variables, has been introduced in biomedicine for numerous applications ranging from epidemiological data to biological data [13]. The simplest way to use MI is to test for a significant association between two variables. Here the null hypothesis is that the two variables are independent. The distribution of the MI under the null hypothesis is (with appropriate scaling) that of a chi-square variable, leading to a significance test for the presence of the association [13]. Cox used MI redundancy to detect a causal nonlinear relation between fast food consumption and risk of campylobacteriosis in women [14]. As a measure of degree of temperature dependence in sex determination, MI has been used to yield insights into evolutionary, environmental and ecological changes [15].

In the bioinformatics field, MI has also been applied to genomics data. Butte and Kohane developed algorithms that reconstruct networks based on MI. The authors inferred that edges exist between gene pairs which have an MI above a certain threshold [16]. More recently, MI and its properties have been applied to eliminate indirect regulatory factors in genome-wide networks. Margolin et al applied MI and the data processing inequality (DPI) to characterize interactions between genes [17]. In their work, DPI was used to determine that there was no direct path between gene_1 _and gene_3 _based on the fact that the MI between gene_1 _and gene_3 _was less than the MI between gene_1 _and gene_2_, and also the MI between gene_2 _and gene_3 _[18].

In this paper we extend our previous work [8,9,19], which establishes associations between clinical entities, and propose an information theoretic approach similar to those described above, which uses MI and DPI to help differentiate between direct and indirect associations, thereby improving our understanding of them.

## Results

### Data statistics

There were 25,074 discharge summaries from New York-Presbyterian Hospital (NYPH) in 2004. After data selection and filtering, there were 1,997 unique drug concepts, 732 unique symptom concepts and 947 unique disease concepts in the database.

### Qualitative evaluation

There were 53 pairs that were found to be associated with the two drugs (Metolazone and Rosiglitazone) and two diseases (heart disease and diabetes). Among them, 27 pairs were randomly selected for qualitative evaluation, which included 14 symptoms associated with the two drugs and 13 symptoms associated with the two diseases. The overall precision was 0.81, where the precision was 0.86 and 0.77 for drug-symptom pairs and disease-symptom pairs, respectively. A subset of the results of the evaluation is shown in Table 1, and the full evaluation set is provided in the supplementary files (Full Drug-Symptom evaluation set available from ; Full Disease-Symptom evaluation set available from ).

**Table 1:** Results of evaluation

**#**	**Association of interest** **MI ((95%CI)**	**Mediators for association of interest** **MI (95%CI)**		Result *(D/I/E)	RS **
	**RX-SX**	**RX-DX**	**DX-SX**		

1	Metolazone-Shortness of Breath *0.086(0.075–0.097)*	Metolazone-Hypertensive Disease *0.526(0.495–0.557)*	Hypertensive Disease-Shortness of Breath *0.132(0.113–0.152*)	I	I
2	Metolazone-Headache *0.070(0.059–0.083*)	Metolazone-Hypertensive Disease *0.526(0.495–0.557)*	Hypertensive Disease-Headache *0.084(0.072–0.095*)	E	E
3	Metolazone-constipation *0.034 (0.023–0.046*)	Metolazone-Hypertensive Disease *0.526(0.495–0.557)*	Hypertensive Disease-constipation *0.019(0.009–0.028*)	E	D
4	Rosiglitazone-Headache *0.079 (0.068–0.091*)	Rosiglitazone-Diabetes *0.443(0.327–0.513)*	Diabetes-Headache *0.117(0.093–0.128*)	I	D
5	Rosiglitazone-Chest Pain *0.077(0.069–0.088)*	Rosiglitazone-Diabetes *0.443(0.327–0.513)*	Diabetes-Chest Pain *0.090(0.074–0.115*)	E	E

	**DX_1 _- SX**	**DX_1 _- DX**_2_	**DX_2 _- SX**		

6	Hypertensive Disease-Shortness of Breath *0.132(0.113–0.152*)	Hypertensive Disease-Heart Failure *0.651(0.563–0.738*)	Heart Failure-Shortness of Breath *0.173(0.158–0.186)*	I	I
7	Diabetes-Chest Pain *0.131(0.115–0.148*)	Diabetes-Heart Failure *0.435(0.317–0.543)*	Heart Failure-Chest Pain *0.184(0.169–0.193)*	I	I

DX: Disease; RX: Drug; SX: Symptom; *I: Indirect association; D; Direct association; E: Either association; ** RS: Reference Standard.

## Discussion

Characterizing clinically important associations based on statistical methods that utilize clinical information extracted from narrative clinical reports is challenging because the associations could correspond to several different relationships. For example, based on clinical knowledge, a drug-symptom association may represent the following relations: 1) a 'treat' relation where the drug is used to treat the symptom (i.e. *Ibuprofen-headache*); 2) a 'cause' relation where the drug causes the symptom (i.e. *Rosiglitazone-headache*); or 3) an 'indirect' relation where the drug treats a disease which is manifested by the symptom (i.e. *Rosiglitazone-polyuria*). Similarly, a disease-symptom association may represent: 1) a 'direct manifestation' where the symptom is a manifestation of the disease (i.e. *diabetes-polyuria*), 2) an 'indirect manifestation' where the symptom is a manifestation of another disease which is a frequent comorbidity of the disease in the association (i.e. *diabetes-angina pectoris*), or 3) a 'treatment-induced' relation where the symptom is caused by a medication or other treatment procedures (i.e. *diabetes-chills*). By applying medical knowledge concerning the possible relations between particular types of entities along with MI and DPI, our findings demonstrated the potential of the approach for differentiating between direct and indirect associations because it achieved a precision of 81%. More interestingly, combining evidence obtained from the approach benefited our understanding of the environmental and phenotypic information in clinical settings. For example, in Figure 1, two sets of evidence were combined to help characterize the association between Metolazone and SOB by joining graphs 1c) and 1d) to generate 1e). In c), the path between Metolazone and SOB was eliminated as was the path between HD and SOB in d). Therefore, a possible path between Metolazone and SOB could be meditated through HD and heart failure.

Upon examination of our results, it appeared that the strengths of the drug-disease/symptom associations can be used to help infer the actual type of relationship. When reviewing the associations and their strengths, we noted that the strong drug-disease/symptom associations were mainly 'treat' relations whereas the weaker drug-disease/symptom associations were either ADEs or 'treat' relations for drugs with broad indications. This observation makes sense clinically and would be interesting to study further using a larger dataset.

Although our results showed that the information theoretic approach could improve our understanding of the associations generated from automated methods, it is, however, still a challenge to accurately identify the nature of associations from patient records, and especially to infer causal links between drug and diseases/symptoms. First, an 'indirect' link based on the DPI method only implies that a 'direct' link has not been proven from the data we have. Therefore, the smallest MI in a triangle does not necessarily imply that there is no direct link. All applications of MI and DPI in characterizing the interdependencies among entities should be understood in this context. Second, determining the correct temporal sequence for environmental and phenotypic events would be helpful in removing confounding relations. Temporally, an ADE must follow the medication event, and a drug treatment event must follow the diagnosis event or symptom event. For example, incorrect 'cause' relations between a drug and a symptom would be eliminated if it were known that the symptom preceded the drug administration. In this paper, we restricted our research to discharge summaries documenting a single hospital stay and to ADEs occurring during that time. We assumed that symptoms associated with a drug could be ADEs of the drug or manifestations of diseases/symptoms the drug treats. Similarly, we assumed that symptoms associated with a disease could be manifestations of that disease or manifestations of diseases highly associated with that disease. Including contextual filters consisting of the sections where the clinical information appears, as described in more detail in the Method section, was important. For drug-disease/symptom associations, preliminary studies showed that a simple strategy without the contextual filters failed to find many ADEs, particularly rare ones. This was likely because symptoms induced by drugs appeared to be highly diluted by those associated with the diseases the drugs treated since the 'treat' relations occurred much more frequently. Our strategy for estimating the temporal sequences was very naïve. We will explore development of more sophisticated temporal models and use of a more comprehensive longitudinal record including outpatient visits and pharmacy orders.

There are a number of other factors affecting precision. One factor is that some symptoms may have been correctly characterized by our method as 'ADEs' but were considered wrong in our evaluation because they have not been discovered yet. Another factor is that another association within the DPI triangle could also be indirect thereby confounding our analysis and demonstrating that our method requires more complexity. For example, our method incorrectly determined that the association between *Rosiglitazone *and *headache *was indirect because it considered the MIs between *Rosiglitazone *and *diabetes*, and *diabetes *and *headache*. However the latter association is also indirect. Based on clinical knowledge we know that diabetes is highly associated with hypertension which includes the manifestation headache. This, however, is not due to a limitation of the method presented in this paper because adjusting all possible confounders in an analysis is beyond the scope of the current study, and is challenging for statistical and information theoretic methods in general. Another problem is due to an information gap that exists in patient records because patient records are fragmented and clinicians may document certain events and ignore others.

Our study had a few limitations. First, the study was restricted to discharge summaries of inpatients only. As a result, our findings should be understood in the context of a sick patient population. However, the same method could be used with outpatient reports, which would include a more balanced population. A second limitation of this investigation is that the evaluation was performed on only 37 associations involving only two drugs and two diseases. A third limitation is that one of the co-authors (XW) generated the reference standard using the reference resources. A more comprehensive evaluation involving a larger sample size and more curators will be undertaken in future work.

## Conclusion

The availability of up-to-date, executable, evidence-based medical knowledge is essential in biomedicine for many applications. Automatic methods, which are aimed at acquiring knowledge from clinical sources, such as clinical reports, have shown promise for constructing and updating practice-based knowledge. In this study, we applied an information theoretic approach involving MI and DPI to improve our understanding of associations consisting of environmental and phenotypic entities occurring in the EHR that were obtained using NLP and statistical methods. The results achieved by the methodology demonstrate its usefulness for generating executable medical knowledge, which can be used in important clinical applications, such as pharmacovigilance.

## Methods

### Materials

The data warehouse in NYPH collects and maintains a variety of structured and unstructured data for patient records. Textual discharge summaries dictated in the year 2004 were de-identified and used in this study after obtaining IRB approval.

### Method framework

**Figure 2:**
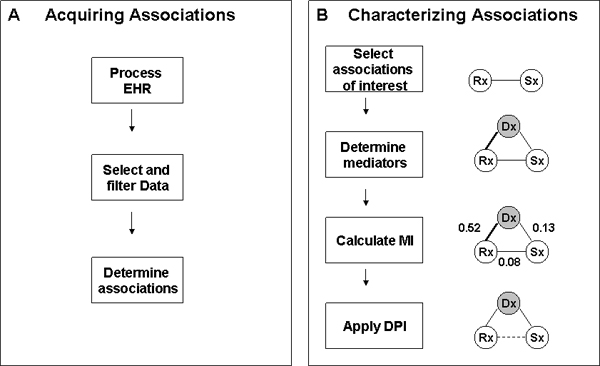
**Schematic diagram of methodological framework**. A: Acquiring Associations; B: Characterizing Associations; EHR: Electric Health Records; MI: Mutual Information; DPI: Data Processing Inequality; Dx: disease; Rx: drug; Sx: symptom.

The framework for detecting environmental and phenotypic associations based on information in narrative reports primarily involves two major phases: acquiring associations and characterizing association, as shown in Figures 2A and 2B.

#### Acquiring associations

There are three steps involved in acquiring associations, which have been described in more detail in [20]. That work aimed to capture adverse drug events occurring during the hospital stay where both the adverse event and the drug therapy were mentioned in the discharge summary. The steps consist of (a) processing the reports using NLP to encode clinical entities; (b) selecting coded entities corresponding to certain environmental and phenotypic properties, and then filtering them to reduce the amount of potentially confounding information, such as negation and eliminating certain sections; (c) applying statistical methods to reveal associations between environmental and phenotypic pairs. The method is briefly described below.

##### a) Process the EHR

MedLEE (the Medical Language Extraction and Encoding System) was used to parse and transform discharge summaries into structured representations consisting of UMLS codes with modifiers. For example, a sentence 'the patient has a headache' is encoded as UMLS code **C0018681** corresponding to *headache*, which has a **certainty **modifier **high certainty **corresponding to *has*.

##### b) Select and filter the data

The semantic classes of the UMLS codes were used to select the appropriate information types for this study, which consisted of two types of phenotypic entities (disease and symptom) and one environmental entity type (drug). For example, the UMLS codes that corresponded to the UMLS semantic classes Disease or Syndrome [T047], Mental or Behavioral Dysfunction [T048], and Neoplastic Process [T191], were used for disease entities. For example, *heart failure *would be selected because it is classified in the UMLS as semantic class T047. The selected phenotypes and drugs were then filtered to exclude entities with specific modifier values, such as family history, certainty modifiers signifying *low *or *no*, and status modifiers signifying past events. Two additional contextual filters consisting of the sections where the clinical information occurred were also applied as a very rough estimation of the appropriate temporal ordering of drug-disease/symptom and disease-symptom events. Heuristically, we know that a symptom occurring during a hospital stay could: 1) be an 'adverse drug event (ADE)' induced by a drug, 2) be a symptom that is an indication for the use of a drug, or 3) be associated with a drug given for a disease or another symptom the drug 'treats'. In order to capture ADEs, we should only consider symptoms that follow the administration of a drug; if a drug is given after the symptom occurs, that drug is unlikely to be the cause of the symptom and more likely to be a treatment. Therefore, drugs mentioned in sections other than Hospital Course and Medications were filtered out in an effort to eliminate medications not given in the hospital, such as Discharge Medications. Drugs in the Medications section were included because typically these contain medications prescribed in outpatient visits prior to admission and continued in the hospital stay, or medications started at admission and continued unless otherwise specified. The second contextual filter filtered out symptoms mentioned in certain sections signifying that they occurred before the hospital stay, such as Chief Complaint and History of Present illness (HPI) since they more frequently describe the disease a patient has than an ADE.

##### c) Determine association tables

We obtained counts for statistical analysis consisting of pairs of co-occurring disease-disease, drug-disease/symptom and disease-symptom as well as counts of relevant types of events. A pair was considered to co-occur if each entity of the pair was selected and not filtered out, and occurred within a single discharge summary. To test the hypothesis of no association between a pair of entities, the χ^2 ^statistic was used. A description of how the cutoff point was determined is described by Cao and colleagues [19,21]. A list was then generated consisting of associations above the cutoff and their strengths.

#### Characterizing associations

The task of characterizing associations between entities as direct or indirect follows four major steps: (a) selecting associations of interest, (b) determining the mediators for associations of interest (c), calculating MI for all associated pairs that are involved, and (d) applying DPI to characterize an association of interest as 'direct', 'indirect' or 'either'.

##### a) Select associations of interest

In this study, we focused on two types of associations: disease-symptom (Dx-Sx) associations and drug-symptom (Rx-Sx) associations.

##### b) Determine mediators for associations of interest

For a drug-symptom association, a mediator was chosen, which was a disease Dx associated with the symptom where the disease had the strongest statistical association with the drug. This was done because the disease was likely to be the treatment indication for the drug, as explained in the Discussion. Thus, the MIs of the two other relevant types of associated pairs (drug-Dx, Dx-symptom) were calculated because they potentially could be indirect paths for the drug-symptom association, as shown in Figures 1a and 1c. Similarly, for a disease_1_-symptom pair, a mediator disease_2_was chosen, which was associated with the symptom and which had the strongest statistical association with disease_1_. MIs for pairs of disease_1_-disease_2 _and the disease_2_-symptom were calculated for similar reasons, as shown in Figures 1b and 1d.

##### c). Calculate Mutual Information (MI)

We calculated the MI following the method proposed by Cover [22]. Given two random variables X and Y within a joint probability mass function p(x, y) and marginal probability mass function p(x) and p(y), the mutual information MI(X; Y) is the relative entropy between joint distribution and the product distribution p(x)p(y), i.e.,



Confidence intervals of MI were calculated by bootstrapping to estimate the variability of the MIs.

##### d) Apply the Data Processing Inequality (DPI)

The DPI states that if random variables X, Y and Z form a Markov chain X-->Y-->Z, then the mutual information between X and Y is greater than or equal to the mutual information between X and Z. That is MI(X; Y) >= MI(X; Z) [17,22,23]. We then used the DPI to characterize the associations for the selected pairs. For example, as illustrated in Figure 1, if a drug Metolazone (ML) and a symptom shortness of breath (SOB) are associated via a disease (i.e., hypertensive disease(HD)), and MI(ML, SOB) <= min [MI(ML, HD); MI(HD, SOB)] and the 95% CIs do not overlap, then we classified the association of the drug-symptom as 'indirect'; if the 95% CIs between the two paths overlapped, the association was classified as 'either', and if neither of those conditions were met, the association was classified as 'direct'.

### Evaluation

We evaluated our methods using a random set of symptoms associated with two drugs of interest, Rosiglitazone and Metolazone, and another random set of symptoms associated with two diseases of interest, hypertensive disease and diabetes.

### Reference standard

Side effects of the two drugs as specified by Micromedex were considered to be 'direct' associations for drug-symptom pairs, while manifestations of the two diseases of interest as specified by WebMD were considered 'direct' associations for disease-symptom pairs [24,25]. In contrast, manifestations of diseases, which the drugs treat, as specified by MicroMedex, were considered to be "indirect" associations for drug-symptom pairs. Similarly, manifestations of other diseases highly associated with either of the two diseases of interest as specified by WebMD were considered as "indirect" associations for disease-symptom pairs. If any of the pairs were determined to be 'direct' and 'indirect', the pair was classified as being 'either'. The reference standard was generated by combining all the 'direct', 'indirect' and 'either' associations for the drugs and diseases.

### Quantitative evaluation

All the selected associations of the two drugs and two diseases were compared to the reference standard. Precision was used to assess the classification performance of our method. In this study precision was measured as the ratio of the number of distinct pairs returned by our method that match the pairs in the reference standard, divided by the total number of pairs found by the method.

## Supplementary material

**Full Drug-Symptom evaluation set **available from http://www.dbmi.columbia.edu/~xiw7002/MI/rx-sx.doc

**Full Disease-Symptom evaluation set **available from http://www.dbmi.columbia.edu/~xiw7002/MI/dx-sx.doc

## Competing interests

The authors declare that they have no competing interests.

## Authors' contributions

XW carried out the study, particularly in the study design, acquisition of data, performing the statistical analysis, drafting and revising the manuscript. GH contributed critically to conception and design of the study and helped to draft the manuscript. CF supervised the overall project and made substantial contributions in study design, coordination, drafting the manuscript and revising it. All authors read and approved the final manuscript.
